# Cardiac dysfunction is attenuated by ginkgolide B via reducing oxidative stress and fibrosis in diabetic rats

**DOI:** 10.22038/ijbms.2020.44210.10358

**Published:** 2020-08

**Authors:** Yu-Xin Jiang, Wei Li, Jing Wang, Guo-Guang Wang

**Affiliations:** 1 School of Medicine, Jiaxing University, Jiaxing, P.R. China; 2 Department of Physiology, Wannan Medical College, Wuhu, P.R. China; 3 Department of Pathophysiology, Wannan Medical College, Wuhu, P.R. China

## Abstract

**Objective(s):**

Diabetic cardiomyopathy is a leading factor of high morbidity and mortality in diabetic patients. Our previous results revealed that ginkgolide B alleviates endothelial dysfunction in diabetic rats. This study aimed to investigate the effect of ginkgolide B on cardiac dysfunction and its mechanism in diabetic rats.

**Materials and Methods:**

Diabetes was induced in rats through the intraperitoneal injection of streptozotocin (STZ). Hemodynamics was monitored to assess cardiac function. Oxidative stress was examined by detecting levels of malondialdehyde (MDA) and superoxide dismutase (SOD) in serum, and expression of sirtuin (SIRT)1, heme oxygenase (HO)-1, and phosphorylated AMPK in the heart. Masson’s trichrome staining and expression of transforming growth factor (TGF)-β1, smooth muscle actin (α-SMA), and phosphorylated (p-) Smad2 and Smad3 were used to evaluate cardiac fibrosis. Inflammatory cytokine in serum and levels of p-PI3K, p-Akt, p-p38, and p-JNK in the heart were determined.

**Results:**

Ginkgolide B significantly improved hemodynamics in diabetic rats. Compared with diabetic rats, treatment with ginkgolide B significantly decreased levels of inflammatory cytokines, improved oxidative stress via reducing MDA concentration, and elevating SOD activity in serum and increasing expression of SIRT1, HO-1, and p-AMPK. Further, ginkgolide B alleviated cardiac fibrosis by decreasing expression of TGF-β1, α-SMA, and p-Smad2 and p-Smad3. Meanwhile, ginkgolide B reduced Levels of p-P38, and p-JNK, and increased levels of p-PI3K and p-Akt.

**Conclusion:**

The results suggested that ginkgolide B alleviated cardiac dysfunction by reducing oxidative stress and cardiac fibrosis.

## Introduction

Diabetes mellitus is a worldwide severe chronic disease. Cardiac failure is a leading factor of high morbidity and mortality in diabetic patients, which is called diabetic cardiomyopathy (DCM) (1). Many vital factors including cardiac hypertrophy, fibrosis, vasculopathy, extracellular matrix changes, and cardiomyocyte apoptosis contribute to the onset and progression of DCM (2). Fibrosis, which results from apoptosis of cardiac cells and excessive production of the extracellular matrix (ECM), plays a vital role in dysfunction of various organs including the heart. It has been widely acknowledged that cardiac fibrosis is involved in the pathogenesis process of DCM, and accelerates the development of DCM (3, 4). Cardiac fibrosis impairs myocardial contractility and the pumping function of the heart, which leads to cardiac dysfunction (5, 6). However, the mechanism causing cardiac fibrosis is still unclear. Oxidative stress is considered the leading cause resulting in myocardial hypertrophy, stiffening, and fibrosis (7). Many studies suggest that hyperglycemia leads to oxidative stress via increasing production of reactive oxygen species (ROS). ROS urges myocardial injury and fibrosis (8).

Transforming growth factor (TGF)-β is a secreted cytokine that is implicated in various cellular functions such as proliferation, migration, and differentiation (9). However, many studies showed that TGF-β is a vital cytokine implicated in fibrogenesis, which is overexpressed and activated in various animal models with fibrotic disease (10, 11). TGF-β is shown to lead to activation of Smad2 and Smad3 through binding to the type II receptor, which accelerates the development of fibrosis (12, 13). Further, TGF-β stimulates myofibroblast differentiation through Rho-GTPase activating mitogen-activated protein kinase (MAPK) pathways (14, 15). Previous studies suggested that MAPK pathways are involved in myocardial fibrosis (16, 17).

Ginkgolide B, a natural terpenoid with bioactivity, is found in ginkgo biloba leaves. It has been confirmed that ginkgolide B could inhibit platelet activation via binding to platelet-activating factor (PAF) receptor, therefore regarded as a natural antagonist of PAF (18, 19). Further studies suggested that ginkgolide B exerts various important pharmacological functions such as antioxidative effect (20), reduction of inflammation (21, 22), and anti-apoptotic effect (23, 24). Recently, researchers demonstrated that ginkgolide B protects human umbilical vein endothelial cells (HUVECs) stimulated by oxidized low-density lipoprotein (25, 26). Our previous study showed that ginkgolide B improves vascular function in diabetic rats (20). However, the cardioprotective effect of ginkgolide B against DCM remains unknown. In the present study, we explored the effect of ginkgolide B on diabetic cardiac dysfunction and its mechanisms in STZ-induced rats.

## Materials and Methods

***Reagents***

Sodium pentobarbital and streptozotocin (STZ) were purchased from Sigma (Sigma Chemical Co., St. Louis, MO, USA). Ginkgolide B and specific ELISA kits for the determination of TNF-α and IL-6 were obtained from Hefei Bomei Biotechnology CO., LTD, (Hefei, China). Rabbit polyclonal antibodies β-actin, TGF-β1, ɑ-SMA, SIRT1, HO-1, AMPK, p-AMPK, p-P38, P38, Smad2, p-Smad2, Smad3, and p-Smad3 were purchased from Abcam (Abcam, Cambridge, MA). Antibodies PI3K, p-PI3K, Akt, p-Akt, p-JNK, JNK, and horseradish peroxidase-conjugated secondary antibody were purchased from Bio Basic Inc. (Canada).

***Animals***

Male *Sprague-Dawley* rats (200–240 g) were obtained from the Experimental Animal Center of Wannan Medical College. All animal experiments were conducted in accordance with Chinese Community guidelines for the use of experimental animals. Animals were raised in a standard animal facility and received a normal diet and water *ad libitum*.

***Diabetic model and experiment design***

The diabetic rat model was prepared as described previously (27). Briefly, diabetes was induced in rats via a single intraperitoneal injection of STZ at 70 mg/kg dose (dissolved in 0.1 mol/l ice-cold sodium citrate buffer, pH 4.5). Age-matched control animals were treated with equivalent volume of solvent. After 72 hr of STZ administration, fasting blood glucose concentration in rats was determined for confirmation of diabetes. The animals were diagnosed as diabetic when blood glucose concentration exceeded 16.7 mmol/l. After diabetes was induced, control and diabetic rats were randomly assigned to three groups: normal control group (NC), diabetes model group (DM), and ginkgolide B treatment group (GT). Rats from NC and DM groups received control food and water; Rats from the DG group received control food and water and were orally administered ginkgolide B (5 mg per Kg body weight).

***Monitoring of hemodynamics***

After treatment with ginkgolide B for 8 weeks, rats were anesthetized with sodium pentobarbital (50 mg/Kg) by intraperitoneal injection. The right carotid artery was separated and a Millarminiature catheter was cannulated into the left ventricle to record systolic pressure (SP), diastolic pressure (DP), and maximal rate of increase/decrease of left ventricle pressure (±*dP/dt*_max_). All parameters were recorded with the MedLab data acquisition system (Nanjing MedEase Co., Nanjing, China).

***Determination of pro-inflammatory cytokines***

Levels of TNF-α and IL-6 in serum were measured using TNF-α and IL-6 specific ELISA commercial kits according to the kit instructions. TNF-α and IL-6 levels in serum were presented as ng/l.

***Estimation of oxidative stress***

Myocardium tissues (10%, w/v) were homogenized in 0.1 mmol/l PBS and centrifuged. SOD activity and MDA level in the supernatant were determined by the appropriate available kits. SOD activity and MDA level were expressed as U/mg protein and nmol/mg protein, respectively.

***Morphological examination***

At the end of the experiment, left ventricle tissues were collected and immersed in 4% formaldehyde. After dehydration, fixed tissues by formaldehyde were embedded in paraffin, and sections were cut at five-μm thickness. Sections were stained with Hematoxylin and Eosin (H&E) for morphological examination and with Masson trichrome for analysis of collagen volume fraction.

***Western blot***

Myocardium tissues were lysed and homogenized in pre-cooled lysis buffer (20 mmol/l Tris-buffered saline, 150 mmol/l NaCl, 1 mmol/l EDTA, 2 mmol/l Na3VO4, 1% Triton X-100, 2 mmol/l PMSF, 2 μg/ml leupeptin, and 2 μg/ml aprotinin). Lysates were centrifugated at 12000 g for 15 min at 4 ^°^C, and proteins in the supernatants were quantified with a BCA kit. Denatured protein in the supernatants was separated by SDS-PAGE and then transferred electrophoretically to nitrocellulose membranes. The membranes were immersed in 5% nonfat milk dissolved in TBS-T buffer (20 mmol/l Tris-HCl, 150 mmol/l NaCl, and 0.1% Tween 20, pH 7.5) to block non-specific sites for 2 hr, and incubated with rabbit polyclonal antibodies β-actin, TGF-β1, ɑ-SMA, Smad2, p-Smad2, Smad3, p-Smad3, JNK, p-JNK, P38, p-P38, PI3K, p-PI3K, SIRT1, HO-1, Akt, and p-Akt (1:500) in TBS-T buffer containing 5% nonfat milk overnight at 4 ^°^C. Then, the membranes were incubated with a secondary HRP-conjugated anti-rabbit antibody (1:10000) in TBS-T for 1 hr. Antigens were determined by visualization with DAB.

***Statistical analysis***

Values are presented as mean ± standard deviation (SD). Statistical analyses were performed with GraphPad Prism (Version 7). Statistical differences were analyzed by unpaired t-test or by one-way analysis of variance (*ANOVA*), followed by Tukey’s* post hoc *analysis. A value of *P*<0.05 was considered to be statistically significant.

## Results

***General characteristics and change of heart weight/ body weight ratio (HW/BW)***

After injection with STZ, rats showed a significant increase in the concentration of fasting blood glucose over experimental time and diabetic features including increase in water consumption and polyuria. Diabetic rats significantly lost body weight compared with NC. However, HW/BW was significantly increased in diabetic rats. Ginkgolide B treatment reduced loss of body weight and HW/BW rate in the diabetic rats (Table 1).

***Effect of ginkgolide B on inflammation***

**Figure 1:**
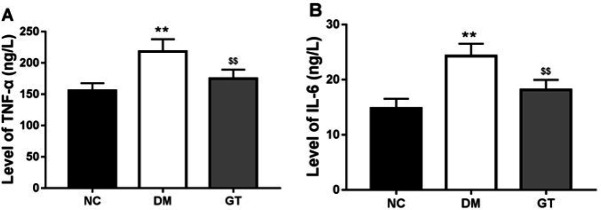
Effects of ginkgolide B on plasma concentrations of pro-inflammatory cytokines. (A) TNF-α concentration. (B) IL-6 concentration. **P-value<0.01 compared with normal control group. $$*P*-value<0.01 compared with diabetes model group. NC: normal control group; DM: diabetes model group; GT: ginkgolide B treatment group; TNF-α: Tumor Necrosis Factor; IL-6: interleukin 6

Hyperglycemia increased levels of inflammatory cytokines such as TNF-α and IL-6 in the serum of STZ-induced diabetic rats (Figures 1A, 1B). In contrast, treatment with ginkgolide B decreased levels of TNF-α and IL-6 (Figures 1A, 1B).

***Effect of ginkgolide B on oxidative stress***

**Figure 2:**
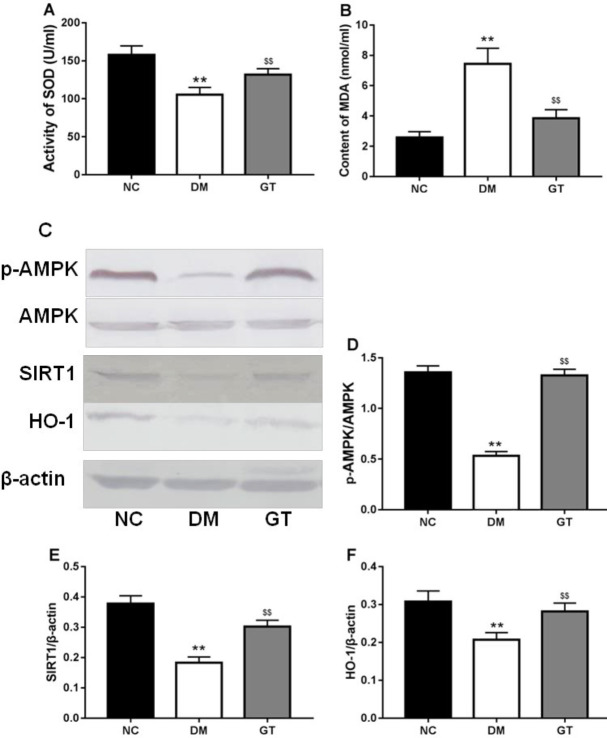
Effects of ginkgolide B on oxidative stress. (A) Activity of SOD. (B) MDA concentration. (C) p-AMPK/SIRT1/HO-1 axis. The relative density of protein expression levels of p- AMPK (D), SIRT1 (E), and HO-1 (F) in the heart analyzed by one-way ANOVA. Significant differences between groups are indicated by symbols (**P-value<0.01 compared with the normal control group and $$*P*-value<0.01 compared with the diabetes model group). NC: normal control group; DM: diabetes model group; GT: ginkgolide B treatment group; SOD: superoxide dismutase; MDA: malondialdehyde; AMPK: AMP-activated protein kinase. SIRT 1: Sirtuin 1; HO-1: Heme Oxygenase-1

It has been well known that oxidative stress plays an important role in diabetic cardiac failure, so we determined the change of anti-oxidation in the myocardium. The results showed that the level of MDA, a product of lipid peroxidation, was significantly increased (Figure 2B), and the activity of SOD was decreased in diabetic rats compared with NC (Figure 2A). Ginkgolide B treatment significantly decreased the level of MDA (Figure 2B) and enhanced activity of SOD (Figure 2A).

Further, diabetes status reduced expression of SIRT1, HO-1, and p-AMPK in the heart, and ginkgolide B treatment significantly restored their expression in diabetic rats (Figure 2).

***Effect of ginkgolide B on pathological changes***

**Figure 3:**
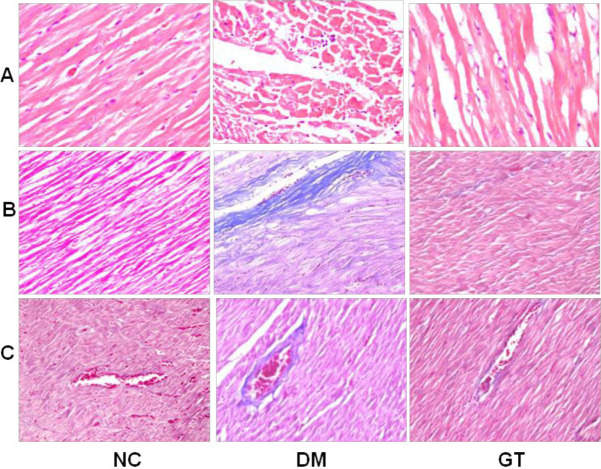
Analysis of sections of myocardial tissues. (A) Haematoxylin and Eosin (H&E) staining. (B) Interstitial fibrosis stained with Masson’s trichrome. (C) perivascular fibrosis stained with Masson’s trichrome. NC: normal control group; DM: diabetes model group; GT: ginkgolide B treatment group

Masson’s trichrome staining showed that cardiomyocytes were well arranged in the NC group, and collagen fibers were rare in the myocardium (Figure 3). Further, collagen fibers in the perivascular and interstitial area were significantly increased in the diabetic rats compared with NC (Figure 3). Ginkgolide B treatment significantly decreased collagen fibers in the perivascular and interstitial area in the ginkgolide B treatment group compared with the diabetes model group (Figure 3).

**Figure 4:**
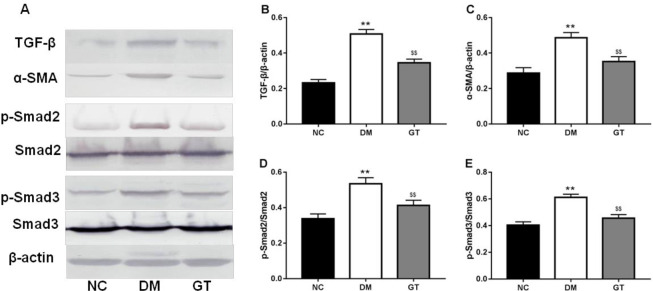
Effects of ginkgolide B on TGF-β/Smads signaling. (A) TGF-β/Smads signaling. The relative density of protein expression levels of TGF-β (B), α-SMA (C), p-Smad2 (D), and p-Smad3 (E) in the heart analyzed by one-way ANOVA. Significant differences between groups are indicated by symbols (**P-value<0.01 compared with the normal control group and $$*P*-value<0.01 compared with the diabetes model group). NC: normal control group; DM: diabetes model group; GT: ginkgolide B treatment group; TGF-β: Transforming growth factor β; SMA: smooth muscle actin

To further estimate the effect of ginkgolide B on cardiac fibrosis in diabetic rats, signaling pathway contributing to cardiac fibrosis was explored. The results revealed that ginkgolide B significantly decreased the expression of TGF-β1, ɑ-SMA, p-Smad2, and p-Smad3 in diabetic rats (Figure 4).

***Improvement of cardiac function by ginkgolide B***

At the end of the experiment, hemodynamic parameters were determined for the assessment of left ventricular performance. The results of the study showed that diabetic rats displayed lower systolic pressure (SP) and diastolic pressure (DP) than NC, and the left ventricular ±*dP/dt*_max_ was significantly depressed in the diabetic rats compared with NC. However, treatment with ginkgolide B lowered the decrease of the parameters including SP, DP, and ±*dP/dt*_max_. Ginkgolide B significantly increased the heart rate of diabetic rats (Table 2).

***Effect of ginkgolide B on TGF-β/MAPKs, PI3K/AKT, and AMPK pathways***

**Figure 5:**
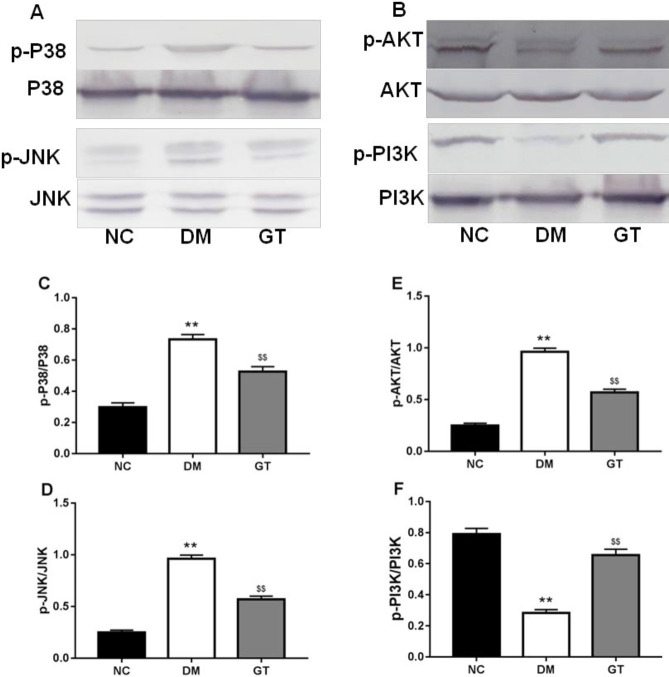
Effects of ginkgolide B on P38/JNK (A) and PI3K/AKT (B) signaling. The relative density of protein expression levels of p- P38 (C), p-JNK (D), p-PI3K (E), and p-AKT (F) in heart analyzed by one-way ANOVA. Significant differences between groups are indicated by symbols (**P-value<0.01 compared with the normal control group and $$*P*-value<0.01 compared with the diabetes model group). NC: normal control group; DM: diabetes model group; GT: ginkgolide B treatment group; JNK: c-Jun N-terminal kinase

The levels of phosphorylated JNK and P38 (p-JNK and p-P38) were increased in the diabetic rats compared with NC (Figure 5). However, ginkgolide B treatment significantly decreased the levels of p-JNK and p-P38 (Figure 4).

Phosphorylated Akt and PI3K were significantly decreased in the DM rats compared with NC (Figure 5). Meanwhile, ginkgolide B treatment elevated phosphorylation of AKT, PI3K, and AMPK in DG (Figure 5).

## Discussion

In the present study, our results showed that treatment with ginkgolide B could improve DCM in diabetic rats. Furthermore, cardioprotective effect of ginkgolide B may be associated with decrease of myocardial fibrosis and improvement of oxidative stress. Our results indicated that ginkgolide B markedly decreased TGF-β1 and ɑ-SMA expression and levels of p-P38, p-Smad2, p-Smad3, and p-JNK in diabetic rats. Ginkgolide B treatment restored reduced expression of SIRT1, HO-1, and p-PMPK by hyperglycemia. In addition, ginkgolide B elevated levels of p-PI3K and p-Akt. The results suggested that ginkgolide B improved cardiac dysfunction via attenuating myocardial fibrosis and reducing inflammation and oxidative stress.

 PI3K/AKT pathway is an important downstream component in the signal transduction of insulin, which is involved in glucose and lipid metabolism (28). Activated PI3K and AKT regulates glucose uptake by translocating glucose transporters to the cell membrane. Further, activated AKT improves myocardial contractility in diabetic mice (29). Down-regulation of PI3K/AKT pathway impaired glucose uptake and led to hyperglycemia in diabetes (30). In the present study, our results showed that ginkgolide B increased activation of PI3K and AKT. 

Oxidative stress has been deemed to play a vital role in the development of diabetes and its complications. Diabetes has been confirmed to reduce the expression of oxidative phosphorylation genes, which are involved in the regulation of expression of antioxidases including SOD, HO-1, and NAD(P)H (31). Sustained hyperglycemia leads to a decrease in antioxidant defenses and increases ROS production. Sirtuin 1 is NAD^+^ -dependent, and implicated in various cellular processes such as stress resistance, survival, cell growth, and metabolism (32). AMPK plays a vital role in regulation of metabolism. The study revealed that activated AMPK has been confirmed to stimulate the translocation of GLUT4 (33). SIRT 1 and AMPK activated PGC-1α via deacetylation and phosphorylation, and improved mitochondrial function (34). Activated PGC-1α induced expression of HO-1, and decreased production of ROS (35). Induction of HO-1 has shown antioxidation and cytoprotective effects in diabetes (36). ROS has been confirmed to be involved in the development and progression of DCM, and contribute to DCM through damaging myocardial cells and remodeling of ECM in the heart (37, 38). The SIRT1-PGC-1α-HO-1 axis is vital in the reduction of oxidative stress by hyperglycemia and protection of the diabetic heart. In addition, Inflammation is one of the important diabetic features (39), and increased inflammation exacerbates DCM in diabetes (40). Oxidative stress resulting from hyperglycemia was confirmed to promote expression of inflammatory cytokines such as TNF-α and IL-6, and generation of ROS accelerates myocardial fibrosis via regulating inflammatory and apoptotic signaling (41). The present study showed that ginkgolide B increased expression of SIRT 1, HO-1, and p-AMPK, elevated SOD activity, decreased MDA content and levels of TNF-α and IL-6.

Transforming growth factor β (TGF-β) is a key growth factor with various biological functions such as stimulation of ROS production, suppression of antioxidant defense, regulation of inflammation, and angiogenesis (42). Evidence demonstrated that TGF-β is implicated in and plays a vital role in the development of various fibrotic diseases, and is considered a profibrogenic cytokine (43). TGF-β mRNA and protein were overexpressed in fibrotic tissue and animal fibrosis model (44). TGF-β could cause phosphorylation of Smad2 and Smad3 in serine residues via binding to the TGF-β type II receptor, the complex of phosphorylated Smad2 and Smad3 with Smad4 translocates to the nucleus and mediates transcription of several genes that are associated with fibrosis (45). The Smad pathway plays a central role in TGF-β-mediated myocardial fibrosis (46). Moreover, TGF-β induced expression of α-smooth muscle actin (α-SMA) by activating Smad 3 (47), and expression of α-SMA is regarded as the hallmark of mature myofibroblasts which secrete excessive ECM proteins (48, 49). Overexpression of ECM is the most important feature of DCM, which can impair the structure and function of the heart (50). In addition, Smad 3 plays an important role in the fibrogenesis pathway induced by TGF-β, and cardiac remodeling is attenuated by decreasing expression of Smad 3 (51). In the present study, our results showed that ginkgolide B treatment decreased expression of TGF-β1 and phosphorylation of Smad 2 and Smad 3.

In addition to Smad signaling, the MAPK pathway is involved in the regulation of the fibrotic process (52). Several studies showed that inhibition of the MAPK pathway alleviated myocardial fibrosis induced by TGF-β2 (53). It has been demonstrated that hyperglycemia can activate MAPK signaling cascades such as ERK1/2, JNK, and p38 MAPK, and activated ERK1/2, JNK, and p38 MAPK modulate the expression of the collagen protein (54). Reduction of ERK1/2 phosphorylation has been confirmed to significantly decrease the expression of collagen I and III mRNA and protein (9). Moreover, inhibition of p38 and JNK reduced expression of PAI-1 mRNA and protein induced by TGF-β1 (55). An experimental study showed that an increase in JNK mRNA expression and activity accelerates the development of myocardial fibrosis in diabetes (16). In the present study, we investigated phosphorylation of p38 and JNK. We found that ginkgolide B attenuated the activity of p38 and JNK.

**Table 1:** Effect of ginkgolide B on BW, HW, and HW/BW ratio in diabetic rats

	Normal control	Diabetes model	Ginkgolide B treatment
BW (g)	418.6 ± 12.2	201.3 ± 10.7**	297.8 ± 16.4^$$^
HW (g)	1.24 ± 0.06	1.05 ± 0.07**	1.10 ± 0.07
HW/BW (mg/g)	2.95 ± 0.15	5.24 ± 0.49**	3.70 ± 0.36^$$^

Values are expressed as mean±SEM. Data were analyzed by unpaired t-test or by one-way ANOVA, followed by Tukey test correction for multiple comparisons. **Represents *P*<0.01, comparison between normal control and diabetes model group, $$ Represents *P*<0.01, comparison between diabetes model and ginkgolide B treatment group. BW: body Weight; HW: Heart Weight; HW/BW: Heart Weight/Body Weight

**Table 2:** Effect of ginkgolide B on left ventricular hemodynamic parameters in diabetic rats

	Normal control	Diabetes model	Ginkgolide B treatment
SP (mmHg)	136.1 ± 6.45	102.4 ± 7.60**	117.5 ± 9.52^$$^
DP (mmHg)	98.6 ± 6.14	67.4 ± 6.09**	83.4 ± 8.14^$$^
+*dP/dt*max (mmHg/s)	4383.1 ± 111.9	3062.3 ± 130.7**	3615.8 ± 127.1^$$^
-*dP/dt*max (mmHg/s)	4377.4 ± 75.1	3079.4 ± 117.7**	3629.6 ± 129.4^$$^
Heart rates (/s)	353 ± 8.65	301 ± 16.58**	332 ± 18.15^$$^

Values are expressed as mean±SEM. Data were analyzed by unpaired t-test or by one-way ANOVA, followed by Tukey test correction for multiple comparisons. **Represents* P*<0.01, comparison between normal control and diabetes model group, $$ Represents *P*<0.01, comparison between diabetes model and ginkgolide B treatment group. SP: Systolic pressure; DP: Diastolic pressure; +dP/dt_max_: maximum rate of rise of left ventricle pressure; -dP/dt_max_: maximum rate of fall of left ventricle pressure

## Conclusion

In summary, the present study showed that ginkgolide B protects against myocardial dysfunction in STZ-induced diabetic rats. Ginkgolide B exerted its beneficial effect by improving oxidative stress and inflammation and attenuating myocardial fibrosis. These findings suggested that ginkgolide B might be a potential drug for the therapy of diabetic myocardial fibrosis.
